# Sex‐Specific Proteomic Profiling Identifies Pregnancy Zone Protein as a Complement‐Linked Marker of Adverse Prognosis in Esophageal Adenocarcinoma

**DOI:** 10.1002/ijc.70687

**Published:** 2026-08-12

**Authors:** Alexander Quaas, Hanna Schiemann, Su Ir Lyu, Thomas Zander, Axel Hillmer, Hans Schlößer, Christiane J. Bruns, Reinhard Büttner, Bastian Grothey

**Affiliations:** ^1^ Institute of Pathology, Faculty of Medicine and University Hospital of Cologne, University of Cologne Cologne Germany; ^2^ Department of Internal Medicine I University Hospital Cologne, Medical Faculty, University of Cologne Cologne Germany; ^3^ Department of General, Visceral and Cancer Surgery, Faculty of Medicine University Hospital of Cologne Cologne Germany

**Keywords:** esophageal adenocarcinoma, pregnancy zone protein, proteomics, sex differences

## Abstract

Esophageal adenocarcinoma (EAC) exhibits marked male predominance with male‐to‐female ratios reaching 8.5:1, yet the molecular basis underlying this sex disparity remains poorly characterized. We analyzed 92 EAC specimens using mass spectrometry–based proteomics, comprising 47 female and 45 male tumors from treatment‐naïve patients. Differential expression, pathway enrichment, immunohistochemical, and survival analyses were used to identify sex‐associated proteomic features and prognostic signatures. Proteomic profiling revealed focal yet biologically meaningful sex differences in EAC. After multiple testing correction, three proteins were differentially expressed: the autosomal protein pregnancy zone protein (PZP), enriched in female tumors, and the Y‐chromosome–encoded proteins DDX3Y and RPS4Y1, which were overexpressed in male tumors. At nominal significance, proteins upregulated in female tumors showed marked enrichment of immune‐related pathways. Proteins correlated with PZP expression formed a network dominated by complement cascade. Clinically, elevated PZP expression was associated with significantly poorer overall survival specifically in male patients, with no significant association detected in the combined or female‐only cohort. Proteome‐wide survival analyses further demonstrated distinct sex‐specific prognostic landscapes. Our study provides the first comprehensive characterization of sex‐associated proteomic differences in EAC. Sex shapes immune‐related pathways, prognostic proteomic signatures, and survival associations. PZP emerges as a sex‐differential, complement‐associated protein with adverse prognostic significance in male patients, highlighting sex as a biologically and clinically relevant variable in EAC.

## Graphical abstract

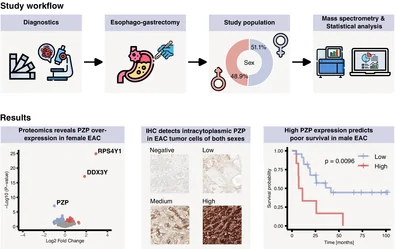

**What's New?**

Although widely observed, the remarkable predominance of esophageal adenocarcinoma (EAC) among males remains poorly understood. To cast light on possible molecular explanations, the authors of the present study performed a comprehensive proteomic analysis of sex‐associated differences in treatment‐naïve EAC, using mass spectrometry to profile more than 6000 proteins. Analyses show that female tumors are enriched in immune‐related pathways. Meanwhile, in males, pregnancy zone protein (PZP) emerged as a novel complement‐associated factor with adverse prognostic significance, highlighting sex‐specific molecular and prognostic landscapes. The findings reinforce sex as an important biological variable in EAC and identify PZP as a potential prognostic marker.

**Abbreviations**
CI: Confidence Intervals
EAC: Esophageal Adenocarcinoma
EDTA: Ethylenediaminetetraacetic acid
FFPE: Formalin Fixation and Paraffin Embedding
FLOT: Fluorouracil, Leucovorin, Oxaliplatin, Docetaxel
HR: Hazard Ratio
IHC: Immunohistochemistry
MCL: Markov Cluster Algorithm
ORA: Over‐Representation Analysis
TMA: Tissue Microarray
TSG: Tumor Suppressor Gene

## 1 Introduction

Esophageal adenocarcinoma (EAC) exhibits one of the most pronounced sex disparities in cancer, with male‐to‐female ratios up to 8.5 in Western populations [1]. This male predominance has persisted despite dramatic increases in EAC incidence over recent decades [2]. While epidemiological factors, including differences in obesity patterns, prevalence erosive gastroesophageal reflux disease, and hormonal influences, have been proposed as contributors, the molecular basis underlying this sex‐specific susceptibility remains poorly understood [2, 3].

Biological sex is increasingly recognized as a critical determinant of cancer initiation, progression, and therapeutic response [4]. Beyond hormone‐driven malignancies, sex differences influence tumor mutational landscapes, immune microenvironment composition, metabolic programs, and clinical outcomes across multiple cancer types [4, 5, 6, 7, 8, 9]. Pan‐cancer studies have revealed sex‐specific patterns in oncogenic signaling, immune checkpoint regulation, and survival, underscoring the importance of sex‐stratified molecular analyses within individual tumor entities [4, 6, 7, 10, 11, 12, 13, 14].

Recent multi‐omic and single‐cell analyses in esophageal cancer have revealed substantial sex‐based biological disparities [10]. The study identified distinct immunological profiles between sexes, including variations in immune cell populations and differential T‐cell exhaustion patterns that correlate with sex‐dependent checkpoint inhibitor responses. Genomic analyses uncovered sex‐associated patterns in mutation frequencies and molecular signatures. However, the study did not distinguish between adenocarcinoma and squamous cell carcinoma subtypes, despite their distinct pathophysiology and molecular differences [10, 15]. A separate genome‐wide association study identified a significant association between the KHDRBS2‐MTRNR2L9 variant and EAC/Barrett's esophagus specifically in males [16].

While these genomic and transcriptomic investigations provide valuable insights, they may not fully capture the complete functional impact of sex‐related molecular variations in EAC. Given that proteins constitute the primary functional effectors of cellular processes and signaling networks, proteomic analysis offers more direct insight into biological phenotypes than nucleic acid‐based approaches. Despite this potential, the proteome‐level characterization of sex‐dependent molecular features in EAC remains limited. Mass spectrometry offers an unbiased platform for comprehensive protein quantification, enabling the characterization of sex‐associated molecular signatures at the functional level.

In this study, we present the first comprehensive proteomic analysis of sex‐associated molecular differences in EAC. Using mass spectrometry–based profiling of over 6000 proteins in a balanced cohort of female and male patients, we identify focal but biologically meaningful sex‐differential protein expression patterns and reveal distinct pathway enrichments across sexes. Among these, pregnancy zone protein (PZP), an α‐macroglobulin family member, emerges as a prominent female‐biased protein linked to complement cascade activity. Additionally, we delineate sex‐specific proteome‐wide survival associations, uncovering markedly different prognostic landscapes in male and female EAC. Our data highlight sex as a biologically consequential variable in EAC and identify PZP as a candidate mediator of sex‐dependent immune biology with potential prognostic significance.

## 2 Methods

### 2.1 Study Design and Patient Cohort

**FIGURE 1:**
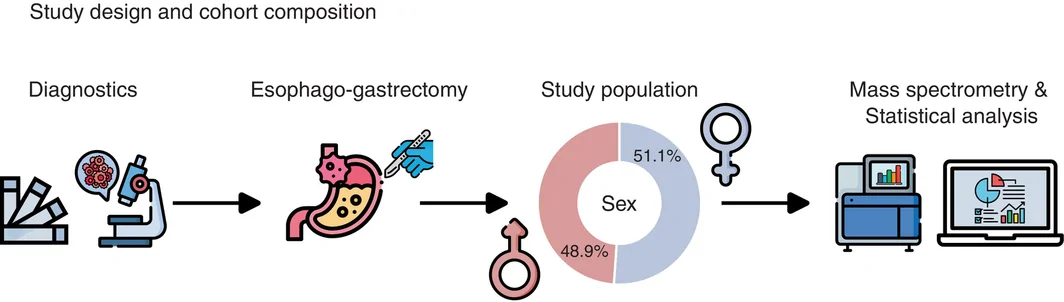
Study design and cohort composition. Schematic representation of the study workflow, from initial diagnosis through proteomic analysis. Following histopathological diagnosis, patients with esophageal adenocarcinoma (EAC) underwent primary esophago‐gastrectomy without neoadjuvant therapy. A cohort of 92 tumors from 47 female (51.1%) and 45 male (48.9%) patients was selected for mass spectrometry‐based proteomic analysis and quantitative characterization of protein expression.

Our retrospective study included 92 patients with adenocarcinoma of the esophagus who underwent primary resection without neoadjuvant treatment at the Department of General, Visceral, Cancer, and Transplantation Surgery of the University Hospital Cologne. Data collection occurred prospectively with subsequent retrospective analysis (Figure 1). Documentation of clinicopathological features followed established protocols based on the Union for International Cancer Control's 7th edition classification system.

Proteomic evaluation using mass spectrometry techniques was conducted on cancerous tissue from the entire patient group (*n* = 92) and additionally on 15 non‐malignant esophageal samples. Quality control identified three female cancer specimens and one normal tissue sample as statistical outliers during mass spectrometry processing, leading to their exclusion. The resulting dataset for proteomic investigation included 89 specimens, distributed between 44 female and 45 male cancer samples. Additional patient characteristics are summarized in Table 1.

**TABLE 1:** Clinicopathological characteristics of patients included in the proteomic analysis cohort. The table presents baseline clinicopathological characteristics of 92 esophageal adenocarcinoma patients selected for mass spectrometry‐based proteomic analysis, stratified by sex. Fisher's exact test was used to assess statistical significance between groups. No significant differences could be observed between female and male EAC patients. pT: Histopathological tumor stage; pN: Histopathological nodal status; L: Lymphovascular invasion; V: Vascular invasion; Pn: Perineural invasion.

Parameter	Total	Sex		Fisher's exact test
		Female	Male	
	*n* (%)	*n* (%)	*n* (%)	*p*
No. of patients	92	47	45	
Age				0.4504
Age (< 65)	24 (26.1)	10 (21.3)	14 (31.1)	
Age (> = 65)	46 (50)	25 (53.2)	21 (46.7)	
Unknown	22 (23.9)	12 (25.5)	10 (22.2)	
pT				0.5534
0	0 (0)	0 (0)	0 (0)	
1	21 (22.8)	10 (21.3)	11 (24.4)	
2	9 (9.8)	3 (6.4)	6 (13.3)	
3	42 (45.7)	24 (51.1)	18 (40)	
4	3 (3.3)	2 (4.3)	1 (2.2)	
Unknown	17 (18.5)	8 (17)	9 (20)	
pN				0.2359
0	26 (28.3)	12 (25.5)	14 (31.1)	
1	25 (27.2)	11 (23.4)	14 (31.1)	
2	13 (14.1)	10 (21.3)	3 (6.7)	
3	11 (12)	6 (12.8)	5 (11.1)	
Unknown	17 (18.5)	8 (17)	9 (20)	
L				0.5434
0	22 (23.9)	14 (29.8)	8 (17.8)	
1	21 (22.8)	11 (23.4)	10 (22.2)	
Unknown	49 (53.3)	22 (46.8)	27 (60)	
V				0.2165
0	49 (53.3)	27 (57.4)	22 (48.9)	
1	2 (2.2)	0 (0)	2 (4.4)	
Unknown	41 (44.6)	20 (42.6)	21 (46.7)	
Pn				0.0894
0	12 (13)	5 (10.6)	7 (15.6)	
1	12 (13)	10 (21.3)	2 (4.4)	
Unknown	68 (73.9)	32 (68.1)	36 (80)	

Immunohistochemical assessment of PZP protein levels utilized tissue microarrays containing 153 EAC specimens (female *n* = 29; male *n* = 124). This collection primarily consisted of samples from individuals who had undergone neoadjuvant treatment before surgical intervention, with a smaller proportion representing primary surgical cases.

Our investigation of sex‐based survival disparities employed our institution's comprehensive database, encompassing outcome data from 95 female and 757 male patients with EAC.

### 2.2 Tissue Microarray Construction and Immunohistochemistry

Tumor samples used for tissue microarray (TMA) generation were processed by formalin fixation and paraffin embedding (FFPE) in accordance with routine histopathological procedures. Experienced pathologists reviewed hematoxylin–eosin–stained sections to select representative tumor regions. From these areas, 1.2‐mm core biopsies were obtained using a semi‐automated precision instrument and subsequently transferred into recipient paraffin blocks to assemble the TMAs. Immunohistochemical analyses were carried out with the Leica BOND‐MAX automated staining platform (Leica Biosystems, Wetzlar, Germany). For PZP detection, we used a rabbit polyclonal antibody (ab233166, Abcam) at 1:500 dilution with Ethylenediaminetetraacetic acid (EDTA) pretreatment.

Cytoplasmic expression of PZP was evaluated using the H‐score system, calculated as follows: H‐Score = 1 × (% cells with weak staining) + 2 × (% cells with moderate staining) + 3 × (% cells with strong staining), yielding scores ranging from 0 to 300 [17, 18]. Based on these values, cases were classified as negative (0–50), weakly positive (51–100), moderately positive (101–200), or strongly positive (201–300). All immunohistochemical assessments were conducted by trained pathologists.

### 2.3 Protein Extraction and Mass Spectrometry Analysis

Protein extraction from FFPE tissue sections and subsequent mass spectrometry analysis were performed according to established protocols. A detailed protocol for sample preparation, mass spectrometry parameters, and data processing is provided in the Supporting Information.

### 2.4 Differential Expression Analysis and Pathway Enrichment

The limma R package was employed to identify differentially expressed proteins between female and male tumor samples [19]. Statistical significance thresholds were set at *p* values below 0.05 and false discovery rate‐adjusted *p* values under 0.2. To investigate biological pathways associated with the protein expression changes, we utilized the ReactomePA package in R, which interfaces with the Reactome pathway database for enrichment analysis [20].

### 2.5 Oncogene and Tumor Suppressor Gene Annotation

Annotation of oncogenes and tumor suppressor genes used in survival analyses were obtained from the OncoKB database (https://www.oncokb.org/).

### 2.6 Statistical Analysis and Data Visualization

Statistical analyses and data visualizations were performed using R version 4.4.2 (R Foundation for Statistical Computing, Vienna, Austria). Protein–protein interaction networks were constructed using the STRING database version 12.0 (https://string‐db.org) with the following parameters: minimum required interaction score of 0.4, interaction partners limited to query proteins only, and network clustering performed using the Markov Cluster Algorithm (MCL) with an inflation parameter of 3 [21]. Graphical illustrations were created using Inkscape (https://inkscape.org/), with supplementary design elements sourced from Flaticon (https://www.flaticon.com).

## 3 Results

### 3.1 Sex‐Related Proteomic Differences in EAC

**FIGURE 2:**
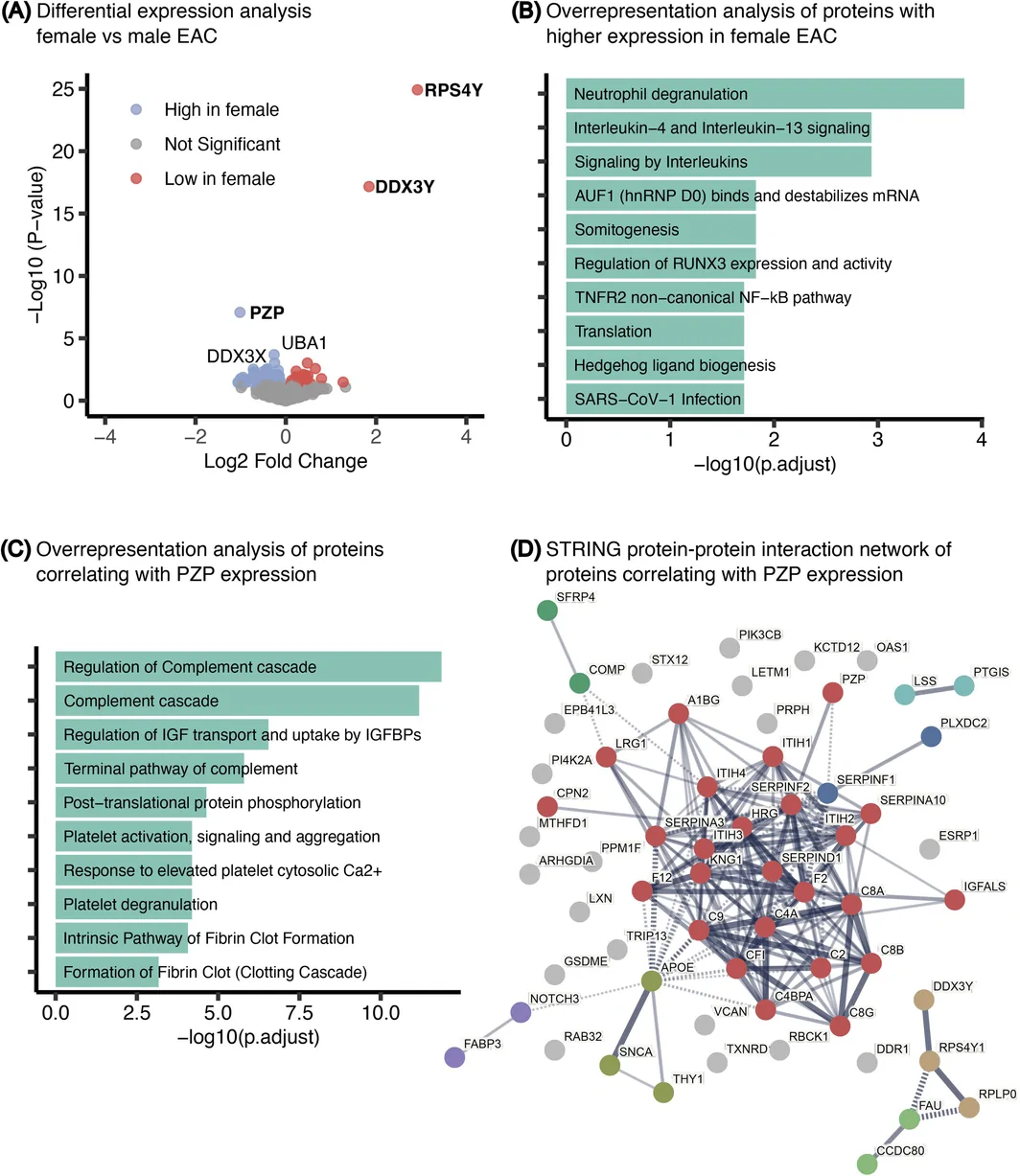
Proteomic characterization of sex‐related differences in esophageal adenocarcinoma. (A) Volcano plot depicting differential protein expression between female and male EAC patients (*n* = 89). Of 6232 quantified proteins, three reached significance after multiple testing correction (*p* < 0.05, adjusted *p* < 0.2; bold labels), while 180 were nominally significant (*p* < 0.05, unadjusted). Blue dots represent proteins enriched in female tumors (negative log2 fold change), red dots indicate proteins enriched in male tumors (positive log2 fold change), and gray dots denote non‐significant changes. (B) Over‐representation analysis (ORA) of proteins upregulated (*p* < 0.05, unadjusted) in female EAC using Reactome pathways. Top 10 enriched pathways are shown, ranked by adjusted *p* value, with neutrophil degranulation and interleukin signaling most significantly enriched. No pathways were significantly enriched for male‐biased proteins. (C) ORA of proteins correlating with PZP expression (|r| > 0.3, *n* = 89) showing enrichment of complement cascade and coagulation‐related pathways. (D) STRING protein–protein interaction network of PZP‐correlated proteins. Node colors indicate MCL clustering; the major cluster (red) comprises complement cascade proteins (C4A, C4BPA, C8A, C8B, C8G, and C9) and coagulation factors, particularly serine protease inhibitors (SERPINF2, SERPINA10, and SERPIND1). Line thickness indicates the strength of data support. Edges between clusters are depicted as dotted lines.

We analyzed a cohort of 92 EAC from patients who underwent primary esophago‐gastrectomy without neoadjuvant therapy (Figure 1). The cohort demonstrated balanced sex distribution with 47 female (51.1%) and 45 male (48.9%) patients. Mass spectrometry‐based proteomic analysis successfully quantified 6232 proteins across 89 evaluable samples. Differential expression analysis between female and male EAC specimens revealed distinct proteomic signatures (Figure 2a; Table S1). While the majority of proteins showed no significant sex‐based differences, three proteins reached statistical significance after multiple testing correction (adjusted *p* < 0.2): PZP (pregnancy zone protein), DDX3Y, and RPS4Y1. As expected, the Y‐chromosome encoded proteins DDX3Y and RPS4Y1 were overexpressed in male tumors. Notably, autosomal protein PZP demonstrated significantly higher expression in female tumors. An additional 180 proteins showed nominal significance (*p* < 0.05, unadjusted), including UBA1, DDX3X, and NOTCH3. Pathway enrichment analysis of nominally significant upregulated proteins in female EAC (*p* < 0.05, unadjusted) identified significant enrichment of immune‐related pathways (Figure 2b; Table S2). Neutrophil degranulation emerged as the most significantly enriched pathway (adjusted *p* < 0.0001), followed by interleukin‐4 and interleukin‐13 signaling, and general interleukin signaling pathways. Additional enriched pathways included AUF1‐mediated mRNA destabilization, somitogenesis, and regulation of RUNX3 expression. In contrast, no pathways reached statistical significance for proteins upregulated in male tumors.

### 3.2 PZP Expression and Complement Cascade Association

Although the Y‐chromosomal proteins DDX3Y and RPS4Y1 showed the strongest differential expression among the three proteins reaching statistical significance after multiple testing correction, PZP emerged as the most biologically relevant candidate for further investigation. Importantly, we observed no reciprocal regulation between these Y‐chromosome proteins and their X‐chromosome homologs (DDX3X and RPS4X) in differential expression analysis between female and male EAC, providing no evidence for compensatory substitution mechanisms. The absence of functional complementation indicates that differential DDX3Y and RPS4Y1 expression simply reflects Y‐chromosome presence in males, with no apparent direct role in tumor biology. We therefore prioritized PZP, as an autosomal protein with active sex‐differential regulation, for comprehensive characterization.

Analysis of proteins correlating with PZP expression in the complete cohort (|r| > 0.3, *n* = 89) revealed strong enrichment for complement cascade and coagulation‐related pathways (Figure 2c; Table S3). The regulation of complement cascade showed the highest enrichment (adjusted *p* = 1*10^‐12), followed by the complement cascade itself, regulation of IGF transport, and terminal pathway of complement. Coagulation‐related pathways, including platelet activation and fibrin clot formation, were also significantly enriched. STRING database protein–protein interaction network analysis of PZP‐correlated proteins revealed a densely connected cluster comprising complement cascade components (C4A, C4BPA, C8A, C8B, C8G, and C9) and serine protease inhibitors (SERPINF2, SERPINA10, and SERPIND1) (Figure 2d; Table S4), suggesting a functional network linking PZP to complement regulation and coagulation processes.

### 3.3 Immunohistochemical Analysis of PZP Expression

**FIGURE 3:**
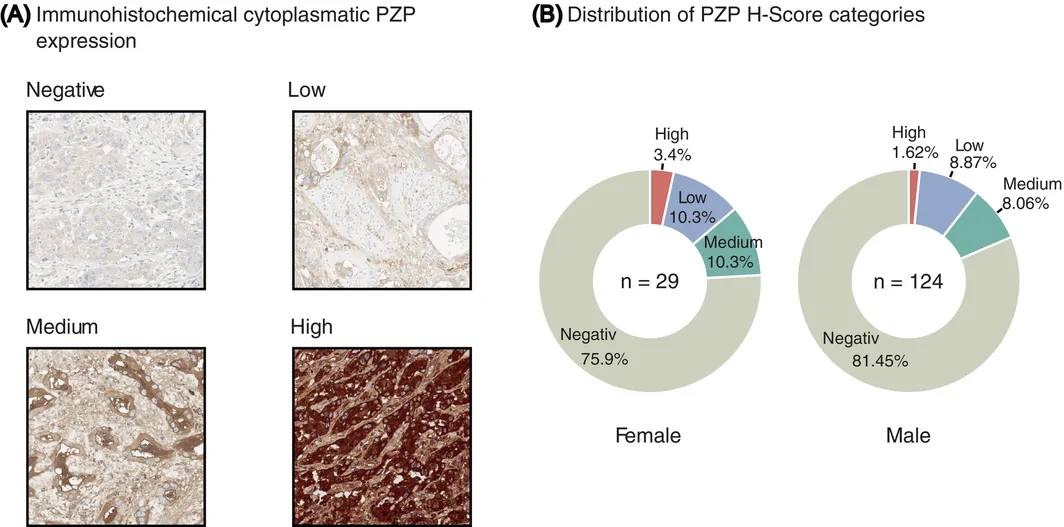
Immunohistochemical validation of PZP expression in esophageal adenocarcinoma. (A) Representative immunohistochemical staining patterns showing cytoplasmic PZP expression across H‐Score intensity categories (negative, low, medium, high). PZP, a plasma protein and member of the α‐macroglobulin family physiologically produced in liver and uterus, exhibits background staining; however, distinct cytoplasmic staining is observed in some tumor cells from both female and male patients. (B) Distribution of PZP H‐score categories in female (*n* = 29) and male (*n* = 124) EAC specimens. The cohort predominantly comprises tumors from patients who received neoadjuvant therapy prior to surgery, with a minority from primary esophago‐gastrectomy. Most tumors showed negative or low expression in both sexes, with few exhibiting medium or high expression. No significant difference in distribution was observed between female and male patients (*p* = 0.69, Fisher's exact test).

Validation of proteomic PZP expression by immunohistochemistry proved challenging, as PZP is a plasma protein physiologically produced, for example, in the liver and uterus, leading to pronounced background stromal staining that complicates quantification of total protein levels. Nevertheless, we investigated whether PZP is actively produced by tumor cells themselves in an extended cohort of 153 EAC specimens on tissue microarrays (29 female and 124 male) (Figure 3). This cohort predominantly comprised patients who received neoadjuvant therapy, in contrast to our proteomics discovery cohort. Interestingly, we observed distinct cytoplasmic PZP staining in tumor cells from both female and male patients, with H‐scores ranging from negative to high intensity (Figure 3a). While quantification of total PZP protein via immunohistochemistry appeared limited due to stromal background, tumor cell‐specific scoring revealed that at least some tumors exhibit medium or high PZP expression in both sexes (Figure 3b). In female patients, 75.9% were negative, 10.3% low, 10.3% medium, and 3.4% highly positive. Male patients showed a similar distribution with 81.45% negative, 8.87% low, 8.06% medium, and 1.6% highly positive, with no significant difference in H‐score distribution between sexes (*p* = 0.69, Fisher's exact test).

### 3.4 PZP Expression and Survival

**FIGURE 4:**
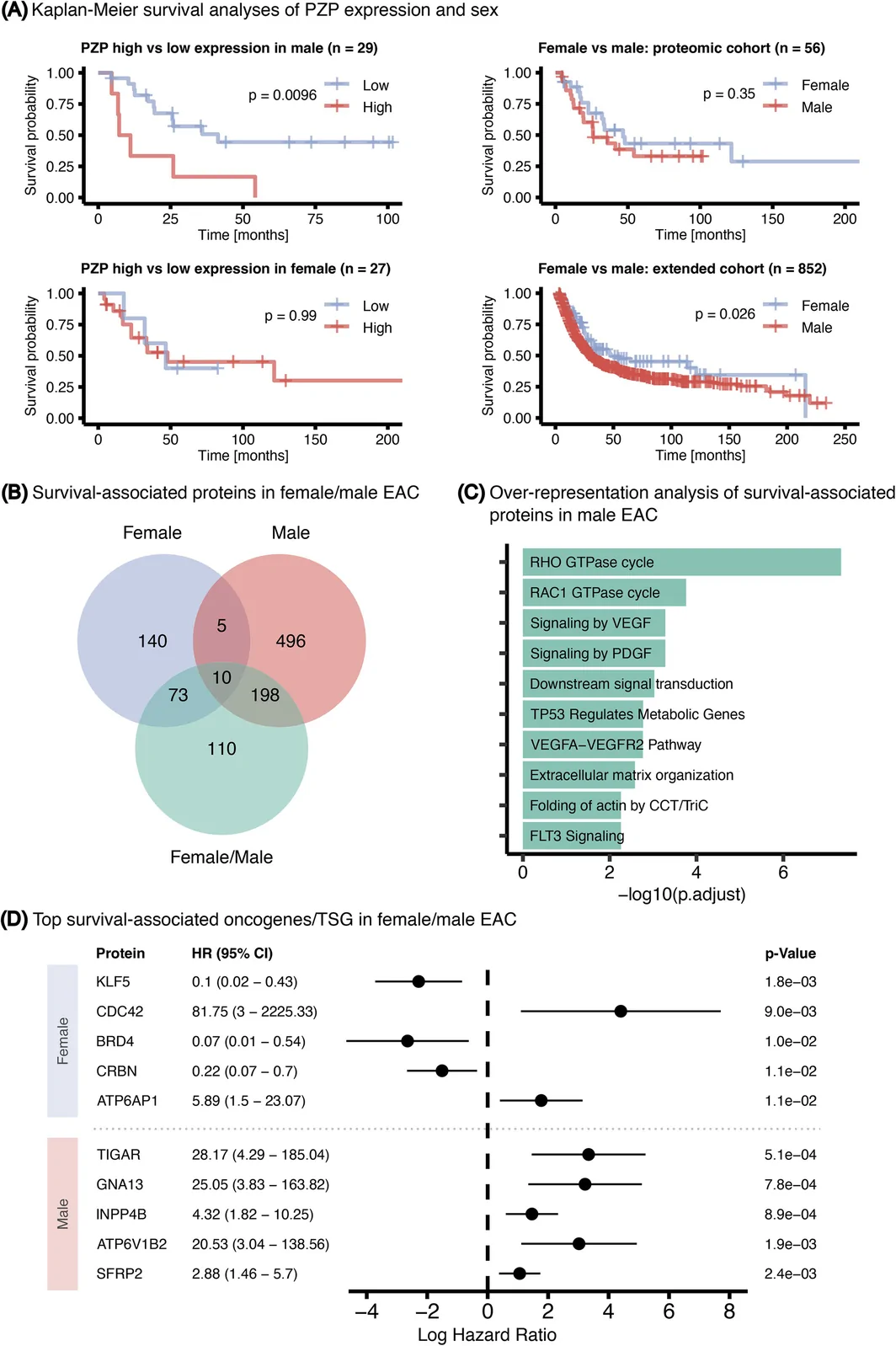
Sex‐specific survival‐associated protein expression patterns in esophageal adenocarcinoma. (A) Kaplan–Meier survival analyses of PZP expression stratified by sex. Left: Patients were stratified by PZP expression using k‐means clustering, yielding PZP‐high (female *n* = 22, male *n* = 6) and PZP‐low (female *n* = 5, male *n* = 23) subgroups. High PZP expression was associated with significantly inferior survival in male patients (top left; *p* = 0.0096), whereas no significant difference was observed in females (bottom left; *p* = 0.99). Overall survival did not differ significantly between female (*n* = 27) and male (*n* = 29) patients in the proteomic cohort (top right; *p* = 0.35), but was significantly superior in females compared to males in the extended institutional cohort (*n* = 852; bottom right; *p* = 0.026). (B) Venn diagram depicting the overlap of survival‐associated proteins identified by univariable Cox regression in female‐only (*n* = 27), male‐only (*n* = 29), and combined (*n* = 56) analyses. Numbers indicate proteins significantly associated with overall survival (*p* < 0.05, unadjusted). (C) Pathway over‐representation analysis of survival‐associated proteins in male EAC, showing the top 10 enriched Reactome pathways (adjusted *p* < 0.05). Enriched pathways include RHO/RAC1 GTPase signaling, VEGF/PDGF signaling, extracellular matrix organization, and TP53‐regulated metabolic gene networks. No significantly enriched pathways were identified for female survival‐associated proteins. (D) Forest plot of the top 5 survival‐associated OncoKB‐annotated oncogenes and tumor suppressor genes (TSGs) identified by univariable Cox regression in female (top; *n* = 27) and male (bottom; *n* = 29) patients (*p* < 0.05, unadjusted). Hazard ratios (HR) with 95% confidence intervals (CI) are shown. Notably, higher expression of the oncogenes KLF5 and BRD4 was associated with favorable survival in females, while in males, elevated TIGAR expression showed the strongest association with inferior survival.

We next evaluated associations between protein expression and patient survival, with particular emphasis on PZP given its differential expression patterns and role in the complement cascade. In univariable Cox regression analyses, higher PZP expression was associated with significantly worse overall survival in male patients (HR = 2.46, *p* = 0.026, *n* = 29), while reaching only borderline significance in the full cohort (HR = 1.71, *p* = 0.0582, *n* = 56, sex‐stratified). No significant association was detected in female patients (HR = 1.25, *p* = 0.4964, *n* = 27). K‐means clustering based on PZP expression in the combined cohort yielded a PZP‐high subgroup (*n* = 28; female *n* = 22, male *n* = 6) and a PZP‐low subgroup (*n* = 28; female *n* = 5, male *n* = 23). Consistent with the Cox regression findings, sex‐stratified survival analysis of these clusters demonstrated significantly inferior survival in PZP‐high compared to PZP‐low male patients (*p* = 0.0096; Figure 4a, top left), whereas no significant difference was observed in female patients (*p* = 0.99; Figure 4a, bottom left). To provide context for the sex‐specific survival association of PZP, we additionally examined overall survival differences between sexes in both cohorts (Figure 4a, right). Within the proteomic cohort, overall survival did not differ significantly between female (*n* = 27) and male (*n* = 29) patients (*p* = 0.35; Figure 4a, top right). In the larger institutional EAC cohort, however, female patients showed significantly better overall survival than male patients (*n* = 852; 95 female, 757 male; *p* = 0.026; Figure 4a, bottom right).

### 3.5 Sex‐Specific Proteome Wide Survival‐Associations

To comprehensively characterize sex‐specific prognostic signatures, we performed univariable Cox regression analysis across the entire proteome. This identified distinct sets of survival‐associated proteins (*p* < 0.05, unadjusted) in sex‐stratified analyses (Figure 4b; Table S5). Female‐only analysis identified 228 proteins uniquely associated with survival, while male‐only analysis identified 709 unique proteins. Notably, only 15 proteins showed consistent survival associations across both sexes.

Pathway analysis of survival‐associated proteins in male EAC revealed enrichment for signaling, cytoskeletal, and extracellular matrix remodeling pathways (Figure 4c; Table S6). The top enriched pathways included RHO GTPase cycle and RAC1 GTPase cycle, followed by growth factor signaling pathways comprising VEGF, VEGFA–VEGFR2, and PDGF signaling, as well as downstream signal transduction. TP53‐regulated metabolic genes, extracellular matrix organization, folding of actin by CCT/TriC, and FLT3 signaling were also significantly enriched. No pathways reached statistical significance for female survival‐associated proteins. Analysis of OncoKB‐annotated oncogenes and tumor suppressors revealed distinct sex‐specific prognostic patterns (Figure 4d). Interestingly, higher expression of the oncogenes KLF5 (HR = 0.1, *p* = 0.0018) and BRD4 (HR = 0.07, *p* = 0.01) were associated with favorable prognosis in females, while increased expression of CDC42 (HR = 81.75, *p* = 0.009) and ATP6AP1 (HR = 5.89, *p* = 0.011) were associated with adverse prognosis. In male patients, TIGAR (HR = 28.17, *p* = 5.1e‐4), GNA13 (HR = 25.05, *p* = 7.8e‐4), and INPP4B (HR = 4.32, *p* = 8.9e‐4) showed the most significant associations with inferior survival, followed by ATP6V1B2 (HR = 20.53, *p* = 0.0019) and SFRP2 (HR = 2.88, *p* = 0.0024).

## 4 Discussion

In this study, we performed the first comprehensive proteomic characterization of sex‐related molecular differences in EAC, analyzing over 6000 proteins in a balanced cohort of female and male patients. Although EAC exhibits one of the strongest male predominances among solid tumors, sex‐specific molecular features have been insufficiently explored [1]. By integrating discovery proteomics, pathway analysis, immunohistochemistry, and survival profiling, we demonstrate that the EAC proteome is shaped by sex‐dependent biology and that these differences have measurable prognostic consequences. Among the proteins identified, pregnancy zone protein (PZP) emerged as the most biologically informative sex‐differentially expressed protein and was further linked to complement regulation and patient survival. These findings underscore that sex is not merely a demographic variable but a biological factor with potential relevance for tumor behavior and clinical trajectories in EAC.

Our proteomic comparison revealed only three proteins reaching significance after correction for multiple testing—PZP, DDX3Y, and RPS4Y1—suggesting that sex‐linked proteomic differences in EAC may be focal rather than global. As anticipated, the Y‐chromosomal proteins DDX3Y and RPS4Y1 were strongly enriched in male tumors, reflecting the presence of the Y chromosome rather than tumor‐intrinsic biology. In contrast, PZP, an autosomal protein with known immunomodulatory functions [22], was more highly expressed in female tumors and thus represented the most biologically meaningful sex‐associated signal. Beyond individual proteins, pathway‐level analyses highlighted pronounced sex differences: proteins upregulated in female tumors demonstrated significant enrichment for immune and inflammatory pathways, including neutrophil degranulation, interleukin‐4/interleukin‐13 signaling, and general interleukin‐mediated pathways. These pathways are consistent with the well‐established phenomenon of stronger innate and adaptive immune responses in females [23]. Conversely, no pathways reached significance in male‐upregulated proteins, suggesting either greater heterogeneity among male tumors or a relative absence of coordinated immune activation. This asymmetry reinforces the concept that female EAC may develop within a more immunologically engaged tumor microenvironment, whereas male tumors may rely more heavily on non‐immune or stress‐responsive biological programs [7, 10, 24, 25]. Given PZP's marked sex bias and biological plausibility, we further investigated its molecular context. Correlation‐based analyses revealed that proteins associated with PZP expression clustered within the complement and coagulation cascades, including C4A, C4BPA, C8A, C8B, C8G, and C9, alongside several serine protease inhibitors. These functional networks align with PZP's known properties as an α‐macroglobulin family member with capacity to bind and inhibit proteases [26]. The identification of PZP at the center of a densely connected complement‐related module suggests that it may participate in shaping the immune milieu of EAC, potentially influencing complement activation, inflammation, and immune evasion.

Immunohistochemical analysis confirmed that PZP is expressed in tumor cells themselves, although evaluation of total protein abundance was limited by physiological background staining inherent to plasma proteins. Nevertheless, the detection of clear cytoplasmic tumor cell–specific staining across both sexes supports the notion that PZP may have tumor‐intrinsic functions rather than representing passive stromal deposition. Although proteomics indicated higher bulk PZP levels in female tumors, tumor cell–specific IHC staining did not differ between sexes. This suggests that tumor cells express comparable amounts of PZP in both sexes, and that the observed sex differences in bulk proteomics may arise from stromal or circulating sources. Alternatively, the predominance of neoadjuvant‐treated patients in the IHC cohort may have attenuated sex‐based differences in PZP expression.

The clinical relevance of these sex‐specific proteomic features is underscored by our survival analyses. Across the entire proteome, we observed a striking disparity: female patients exhibited 228 survival‐associated proteins, whereas male patients exhibited 709, implying that male tumors possess a far more survival‐informative proteomic landscape. Male‐specific survival‐associated proteins were enriched for pathways associated with diverse forms of cellular stress adaptation, including RHO/RAC1 GTPase‐mediated responses to genotoxic and endoplasmic reticulum stress, hypoxia‐ and oxidative stress‐driven growth factor signaling (VEGF, VEGFA–VEGFR2, and PDGF), mechanotransduction through extracellular matrix remodeling, and TP53‐regulated redox and metabolic homeostasis [27, 28, 29, 30, 31, 32, 33, 34, 35, 36, 37]. These findings align with accumulating evidence that male tumor carcinogenesis depends more heavily on stress‐responsive biological programs, as discussed above [7, 24, 25].

Within this broader context, PZP emerged as a prognostically relevant marker with a striking sex‐specific pattern. Although PZP expression was significantly higher in female tumors, elevated tumor PZP expression was associated with significantly worse overall survival exclusively in male patients, while the association was absent in females and reached only borderline significance in the combined cohort. K‐means clustering reinforced this finding: PZP‐high male patients experienced markedly inferior outcomes compared to PZP‐low males, whereas no survival difference was observed in females regardless of cluster assignment. Notably, the PZP‐high cluster was predominantly composed of female patients, consistent with the higher baseline PZP expression in female tumors. This paradoxical pattern, with a protein physiologically enriched in females exerting prognostic impact exclusively in males, suggests that PZP elevation in male tumors represents an aberrant upregulation that may impose immunosuppressive pressure through complement dysregulation in an already less immunologically engaged microenvironment.

Beyond PZP, sex‐stratified survival analysis of OncoKB‐annotated oncogenes and tumor suppressors revealed marked differences in prognostic patterns between male and female EAC. In female patients, BRD4 and KLF5 emerged as strongly protective, suggesting that higher expression of these transcriptional regulators may reflect a less aggressive tumor phenotype in female EAC. CDC42 and ATP6AP1 were conversely associated with adverse prognosis in females. In male patients, TIGAR, GNA13, and ATP6V1B2 showed the strongest associations with inferior survival, alongside INPP4B and SFRP2, pointing to dysregulated metabolic, signaling, and lysosomal pathways as drivers of poor outcomes in male EAC [38, 39, 40, 41, 42].

This study has some limitations. The sample size, although substantial for proteomic profiling, becomes modest when stratified by sex, particularly for survival analyses. Functional experiments were beyond the scope of this work, limiting mechanistic conclusions regarding PZP's role in complement regulation or tumor progression. The IHC validation cohort included many patients who received neoadjuvant therapy, introducing potential treatment‐related confounding. Moreover, proteomic correlations remain associative and require experimental validation to delineate causal pathways.

Future research should focus on validating PZP as a prognostic biomarker in larger, independent cohorts with balanced sex representation. Mechanistic interrogation of PZP's interactions with complement proteins and hormonal regulation may elucidate how this protein shapes tumor–immune dynamics. Sex‐specific prognostic models incorporating proteomic markers, including PZP, could enhance clinical risk stratification. Given renewed interest in targeting complement pathways pharmacologically, understanding sex‐dependent complement biology in EAC may open new therapeutic opportunities.

## 5 Conclusions

Our study provides the first comprehensive proteomic characterization of sex‐related differences in EAC. Female tumors were enriched for immune‐related pathways, while male tumors showed broader survival‐informative proteomic landscapes linked to cytoskeletal, growth factor signaling, and extracellular matrix programs. PZP, overexpressed in female tumors, emerged as a key sex‐differential and prognostically relevant marker, with high expression predicting poorer outcomes specifically in male patients. These findings highlight the importance of sex as a biological variable in EAC and suggest that incorporating sex‐specific proteomic markers could refine prognostic models and guide personalized therapies. Future research should aim at a more detailed functional characterization of PZP, particularly its role in complement regulation, and at validating its prognostic value in larger, independent cohorts.

## Author Contributions

**Alexander Quaas:** conceptualization, data curation, writing – review and editing, supervision, resources, funding acquisition, project administration. **Hanna Schiemann:** conceptualization, writing – review and editing, data curation. **Su Ir Lyu:** writing – review and editing. **Thomas Zander:** writing – review and editing. **Axel Hillmer:** writing – review and editing. **Hans Schlößer:** writing – review and editing. **Christiane J. Bruns:** writing – review and editing. **Reinhard Büttner:** writing – review and editing, resources. **Bastian Grothey:** conceptualization, writing – original draft, methodology, visualization, validation, writing – review and editing, software, formal analysis, project administration, data curation, supervision, investigation.

## Funding

Proteomic analysis was performed by the CECAD Proteomics Facility, CECAD—Cluster of Excellence, University of Cologne, using a Q‐Exactive Exploris 480 LC MS system (INST 1856/71‐1 FUGG) funded by the Deutsche Forschungsgemeinschaft.

## Ethics Statement

The study was approved by the Ethics Committee of the University Hospital Cologne (reference number protocol code 20–1393) and conducted in accordance with the Declaration of Helsinki. Informed consent was obtained from all individual participants included in the study.

## Conflicts of Interest

The authors declare no conflicts of interest.

## Supporting information


**Table S1:** Detailed results of differential expression analysis (female vs. male EAC).
**Table S2:** Detailed results from Reactome pathway overrepresentation analyses of proteins that showed nominal significant higher expression in female versus male EAC.
**Table S3:** Detailed results from Reactome pathway overrepresentation analyses of proteins correlating with PZP expression in the complete cohort.
**Table S4:** Detailed results and color annotation from STRING DB MCL clustering of proteins correlating with PZP expression in the complete cohort.
**Table S5:** Cox regression analysis of protein expression and survival in different subsets of EAC patients.
**Table S6:** Detailed results from Reactome pathway overrepresentation analyses of proteins that showed significant associations between protein expression levels and survival in male EAC patients.

## Data Availability

The mass spectrometry proteomics data have been deposited to the ProteomeXchange Consortium via the PRIDE partner repository with the dataset identifier annotations PXD078617 [43, 44]. The source code, preprocessed mass spectrometry data and clinicopathological annotations are publicly available on Zenodo (https://doi.org/10.5281/zenodo.20261062). Further data that support the findings of this study are available from the corresponding author upon request.

## References

[1] M. Arnold , I. Soerjomataram , J. Ferlay , and D. Forman , “Global Incidence of Oesophageal Cancer by Histological Subtype in 2012,” Gut 64, no. 3 (2015): 381–387, 10.1136/gutjnl-2014-308124. 25320104

[2] S. H. Xie and J. Lagergren , “The Male Predominance in Esophageal Adenocarcinoma,” Clinical Gastroenterology and Hepatology 14, no. 3 (2016): 338–347, 10.1016/j.cgh.2015.10.005. 26484704

[3] M. Rutegård , P. Lagergren , H. Nordenstedt , and J. Lagergren , “Oesophageal Adenocarcinoma: The New Epidemic in Men?,” Maturitas 69, no. 3 (2011): 244–248, 10.1016/j.maturitas.2011.04.003. 21602001

[4] S. Haupt , F. Caramia , S. L. Klein , J. B. Rubin , and Y. Haupt , “Sex Disparities Matter in Cancer Development and Therapy,” Nature Reviews. Cancer 21, no. 6 (2021): 393–407, 10.1038/s41568-021-00348-y. PMC8284191 33879867

[5] S. Anbarasu and A. Anbarasu , “Cancer‐Biomarkers Associated With Sex Hormone Receptors and Recent Therapeutic Advancements: A Comprehensive Review,” Medical Oncology 40, no. 6 (2023): 171, 10.1007/s12032-023-02044-3. 37162589

[6] C. H. Li , S. D. Prokopec , R. X. Sun , et al., “Sex Differences in Oncogenic Mutational Processes,” Nature Communications 11, no. 1 (2020): 4330, 10.1038/s41467-020-17359-2. PMC7455744 32859912

[7] J. Han , Y. Yang , X. Li , et al., “Pan‐Cancer Analysis Reveals Sex‐Specific Signatures in the Tumor Microenvironment,” Molecular Oncology 16, no. 11 (2022): 2153–2173, 10.1002/1878-0261.13203. PMC9168759 35229456

[8] Y. Cai , N. J. W. Rattray , Q. Zhang , et al., “Sex Differences in Colon Cancer Metabolism Reveal A Novel Subphenotype,” Scientific Reports 10, no. 1 (2020): 4905, 10.1038/s41598-020-61851-0. PMC7078199 32184446

[9] F. Mauvais‐Jarvis , N. Bairey Merz , P. J. Barnes , et al., “Sex and Gender: Modifiers of Health, Disease, and Medicine,” Lancet 396, no. 10250 (2020): 565–582, 10.1016/S0140-6736(20)31561-0. PMC7440877 32828189

[10] H. Yan , J. Huang , Y. Li , and B. Zhao , “Sex Disparities Revealed by Single‐Cell and Bulk Sequencing and Their Impacts on the Efficacy of Immunotherapy in Esophageal Cancer,” Biology of Sex Differences 15, no. 1 (2024): 22, 10.1186/s13293-024-00598-z. PMC10941500 38491510

[11] J. Li , Z. Lan , W. Liao , et al., “Histone Demethylase KDM5D Upregulation Drives Sex Differences in Colon Cancer,” Nature 619, no. 7970 (2023): 632–639, 10.1038/s41586-023-06254-7. PMC10529424 37344599

[12] Y. Yuan , L. Liu , H. Chen , et al., “Comprehensive Characterization of Molecular Differences in Cancer Between Male and Female Patients,” Cancer Cell 29, no. 5 (2016): 711–722, 10.1016/j.ccell.2016.04.001. PMC4864951 27165743

[13] H. Li , W. Jiang , S. Liu , et al., “Connecting the Mechanisms of Tumor Sex Differences With Cancer Therapy,” Molecular and Cellular Biochemistry 479, no. 2 (2024): 213–231, 10.1007/s11010-023-04723-1. 37027097

[14] J. A. Pinto , J. M. Araujo , and H. L. Gómez , “Sex, Immunity, and Cancer,” Biochimica et Biophysica Acta (BBA)‐Reviews on Cancer 1877, no. 1 (2022): 188647, 10.1016/j.bbcan.2021.188647. 34767966

[15] H. Yang , F. Wang , C. L. Hallemeier , T. Lerut , and J. Fu , “Oesophageal Cancer,” Lancet 404, no. 10466 (2024): 1991–2005, 10.1016/S0140-6736(24)02226-8. 39550174

[16] J. Dong , C. Maj , S. Tsavachidis , et al., “Sex‐Specific Genetic Associations for Barrett's Esophagus and Esophageal Adenocarcinoma,” Gastroenterology 159, no. 6 (2020): 2065–2076, 10.1053/j.gastro.2020.08.052. PMC9057456 32918910

[17] K. S. McCarty , L. S. Miller , E. B. Cox , J. Konrath , and K. S. McCarty , “Estrogen Receptor Analyses. Correlation of Biochemical and Immunohistochemical Methods Using Monoclonal Antireceptor Antibodies,” Archives of Pathology & Laboratory Medicine 109, no. 8 (1985): 716–721. 3893381

[18] H. Goulding , S. Pinder , P. Cannon , et al., “A New Immunohistochemical Antibody for the Assessment of Estrogen Receptor Status on Routine Formalin‐Fixed Tissue Samples,” Human Pathology 26, no. 3 (1995): 291–294, 10.1016/0046-8177(95)90060-8. 7890280

[19] M. E. Ritchie , B. Phipson , D. Wu , et al., “Limma Powers Differential Expression Analyses for RNA‐Sequencing and Microarray Studies,” Nucleic Acids Research 43, no. 7 (2015): e47, 10.1093/nar/gkv007. PMC4402510 25605792

[20] G. Yu and Q. Y. He , “ReactomePA: An R/Bioconductor Package for Reactome Pathway Analysis and Visualization,” Molecular BioSystems 12, no. 2 (2016): 477–479, 10.1039/c5mb00663e. 26661513

[21] D. Szklarczyk , R. Kirsch , M. Koutrouli , et al., “The STRING Database in 2023: Protein‐Protein Association Networks and Functional Enrichment Analyses for Any Sequenced Genome of Interest,” Nucleic Acids Research 51, no. D1 (2023): D638–D646, 10.1093/nar/gkac1000. PMC9825434 36370105

[22] J. Huang , Y. Xu , Y. Chen , et al., “Revisiting the Role of Pregnancy Zone Protein (PZP) as a Cancer Biomarker in the Immunotherapy Era,” Journal of Translational Medicine 22, no. 1 (2024): 500, 10.1186/s12967-024-05321-5. PMC11128099 38797856

[23] S. L. Klein and K. L. Flanagan , “Sex Differences in Immune Responses,” Nature Reviews. Immunology 16, no. 10 (2016): 626–638, 10.1038/nri.2016.90. 27546235

[24] S. Haupt , F. Caramia , A. Herschtal , et al., “Identification of Cancer Sex‐Disparity in the Functional Integrity of p53 and Its X Chromosome Network,” Nature Communications 10, no. 1 (2019): 5385, 10.1038/s41467-019-13266-3. PMC6879765 31772231

[25] A. Allegra , S. Caserta , S. Genovese , G. Pioggia , and S. Gangemi , “Gender Differences in Oxidative Stress in Relation to Cancer Susceptibility and Survival,” Antioxidants 12, no. 6 (2023): 1255, 10.3390/antiox12061255. PMC10295142 37371985

[26] E. L. Skornicka , N. Kiyatkina , M. C. Weber , M. L. Tykocinski , and P. H. Koo , “Pregnancy Zone Protein Is a Carrier and Modulator of Placental Protein‐14 in T‐Cell Growth and Cytokine Production,” Cellular Immunology 232, no. 1–2 (2004): 144–156, 10.1016/j.cellimm.2005.03.007. 15882859

[27] M. D. Bright , P. A. Clarke , P. Workman , and F. E. Davies , “Oncogenic RAC1 and NRAS Drive Resistance to Endoplasmic Reticulum Stress Through MEK/ERK Signalling,” Cellular Signalling 44 (2018): 127–137, 10.1016/j.cellsig.2018.01.004. PMC6562199 29329780

[28] C. Bailly , C. Degand , W. Laine , V. Sauzeau , and J. Kluza , “Implication of Rac1 GTPase in Molecular and Cellular Mitochondrial Functions,” Life Sciences 342 (2024): 122510, 10.1016/j.lfs.2024.122510. 38387701

[29] X. Wang , D. Liu , F. Wei , et al., “Stress‐Sensitive Protein Rac1 and Its Involvement in Neurodevelopmental Disorders,” Neural Plasticity 2020 (2020): 8894372, 10.1155/2020/8894372. PMC7707960 33299404

[30] B. L. Krock , N. Skuli , and M. C. Simon , “Hypoxia‐Induced Angiogenesis: Good and Evil,” Genes & Cancer 2, no. 12 (2011): 1117–1133, 10.1177/1947601911423654. PMC3411127 22866203

[31] M. González‐Rubio , S. Voit , D. Rodríguez‐Puyol , M. Weber , and M. Marx , “Oxidative Stress Induces Tyrosine Phosphorylation of PDGF Alpha‐and Beta‐Receptors and pp60c‐Src in Mesangial Cells,” Kidney International 50, no. 1 (1996): 164–173, 10.1038/ki.1996.299. 8807585

[32] J. K. Salabei , T. D. Cummins , M. Singh , S. P. Jones , A. Bhatnagar , and B. G. Hill , “PDGF‐Mediated Autophagy Regulates Vascular Smooth Muscle Cell Phenotype and Resistance to Oxidative Stress,” Biochemical Journal 451, no. 3 (2013): 375–388, 10.1042/BJ20121344. PMC4040966 23421427

[33] R. Mallick , A. B. Montaser , H. Komi , et al., “VEGF‐B Is a Novel Mediator of ER Stress Which Induces Cardiac Angiogenesis via RGD‐Binding Integrins Independent of VEGFR1/NRP Activities,” Molecular Therapy 33, no. 7 (2025): 3242–3256, 10.1016/j.ymthe.2025.03.012. PMC12265967 40083161

[34] J. Winkler , A. Abisoye‐Ogunniyan , K. J. Metcalf , and Z. Werb , “Concepts of Extracellular Matrix Remodelling in Tumour Progression and Metastasis,” Nature Communications 11, no. 1 (2020): 5120, 10.1038/s41467-020-18794-x. PMC7547708 33037194

[35] J. M. Northcott , I. S. Dean , J. K. Mouw , and V. M. Weaver , “Feeling Stress: The Mechanics of Cancer Progression and Aggression,” Frontiers in Cell and Developmental Biology 6 (2018): 17, 10.3389/fcell.2018.00017. PMC5835517 29541636

[36] J. Zhuang , W. Ma , C. U. Lago , and P. M. Hwang , “Metabolic Regulation of Oxygen and Redox Homeostasis by p53: Lessons From Evolutionary Biology?,” Free Radical Biology & Medicine 53, no. 6 (2012): 1279–1285, 10.1016/j.freeradbiomed.2012.07.026. PMC3444283 22841759

[37] L. Jiang , J. H. Hickman , S. J. Wang , and W. Gu , “Dynamic Roles of p53‐Mediated Metabolic Activities in ROS‐Induced Stress Responses,” Cell Cycle 14, no. 18 (2015): 2881–2885, 10.1080/15384101.2015.1068479. PMC4825584 26218928

[38] J. Tang , L. Chen , Z. H. Qin , and R. Sheng , “Structure, Regulation, and Biological Functions of TIGAR and Its Role in Diseases,” Acta Pharmacologica Sinica 42, no. 10 (2021): 1547–1555, 10.1038/s41401-020-00588-y. PMC8463536 33510458

[39] T. Worzfeld , N. Wettschureck , and S. Offermanns , “G(12)/G(13)‐Mediated Signalling in Mammalian Physiology and Disease,” Trends in Pharmacological Sciences 29, no. 11 (2008): 582–589, 10.1016/j.tips.2008.08.002. 18814923

[40] Y. Yuan , J. Zhang , Q. Chang , et al., “De Novo Mutation in ATP6V1B2 Impairs Lysosome Acidification and Causes Dominant Deafness‐Onychodystrophy Syndrome,” Cell Research 24, no. 11 (2014): 1370–1373, 10.1038/cr.2014.77. PMC4220163 24913193

[41] N. Srivastava , R. Sudan , and W. G. Kerr , “Role of Inositol Poly‐Phosphatases and Their Targets in T Cell Biology,” Frontiers in Immunology 4 (2013): 288, 10.3389/fimmu.2013.00288. PMC3779868 24069021

[42] R. Surana , S. Sikka , W. Cai , et al., “Secreted Frizzled Related Proteins: Implications in Cancers,” Biochimica et Biophysica Acta. BBA, Reviews on Cancer 1845, no. 1 (2014): 53–65, 10.1016/j.bbcan.2013.11.004. 24316024

[43] E. W. Deutsch , N. Bandeira , Y. Perez‐Riverol , et al., “The ProteomeXchange Consortium in 2026: Making Proteomics Data FAIR,” Nucleic Acids Research 54, no. D1 (2026): D459–D469, 10.1093/nar/gkaf1146. PMC12807779 41206473

[44] Y. Perez‐Riverol , C. Bandla , D. J. Kundu , et al., “The PRIDE Database at 20 Years: 2025 Update,” Nucleic Acids Research 53, no. D1 (2025): D543–D553, 10.1093/nar/gkae1011. PMC11701690 39494541

