# Supplementary material for: Sex‐Specific Proteomic Profiling Identifies Pregnancy Zone Protein as a Complement‐Linked Marker of Adverse Prognosis in Esophageal Adenocarcinoma
Source: Int J Cancer. 2026 Aug 12;159(10):2595–605. doi: 10.1002/ijc.70687 (PMC13595454; doi:10.1002/ijc.70687)
Supplement: Supplementary file 1 [file IJC-159-2595-s001.pdf]

Sex-specific proteomic profiling identifies pregnancy zone protein as a complement-linked marker of adverse prognosis in esophageal adenocarcinoma

Alexander Quaas, Hanna Schiemann, Su Ir Lyu, Thomas Zander, Axel Hillmer, Hans Schloesser, Christiane J. Bruns, Reinhard Büttner, Bastian Grothey

Table of Contents

Supplementary Methods

Protein Extraction and Mass Spectrometry Analysis Page 2 - 3

Supplementary References Page 4

Supplementary Tables (available in separate file: Supplementary_data.xlsx)

Supplementary Table S1. Detailed results of differential expression analysis (female vs male EAC). Sheet 1

Supplementary Table S2. Detailed results from Reactome pathway overrepresentation analyses of proteins that showed nominal significant higher expression in female vs male EAC. Sheet 2

Supplementary Table S3. Detailed results from Reactome pathway overrepresentation analyses of proteins correlating with PZP expression in the complete cohort. Sheet 3

Supplementary Table S4. Detailed results and color annotation from STRING DB MCL clustering of proteins correlating with PZP expression in the complete cohort. Sheet 4

Supplementary Table S5. Cox regression analysis of protein expression and survival in different subsets of EAC patients. Sheet 5

Supplementary Table S6. Detailed results from Reactome pathway overrepresentation analyses of proteins that showed significant associations between protein expression levels and survival in male EAC patients. Sheet 6

Protein Extraction and Mass Spectrometry Analysis

1. FFPE-Tissue Lysis and digestion

FFPE tissue scrolls (roughly 100mm^2 x 5 µm) were transferred to 100 µl tissue lysis buffer (TLB, Covaris) in an AFA-TUBE TPX 96 well Plate (Covaris). After sealing with strip caps, the plates were incubated at 95°C for 10 min in a thermocycler. For subsequent deparaffinization plates were transferred to a Covaris LE220-plus Focused-ultrasonicator and processed with 450 W peak power, 50% duty cycle and 200 cycles at 20°C for 5 min. To de-crosslink proteins, the samples were subsequently incubated at 95° for 90 min in a thermocycler and proteins were finally extracted by a second ultrasonication step in the Covaris LE220 using the settings described above. TCEP and chloroacetamide were added to final concentrations of 5 mM and 20 mM respectively to reduced disulfide bonds and alkylate cysteines. Samples were incubated for 20 min at 55°C in a Thermocycler. 50 µl of protein lysates were used for protein digestion following a modifies version of the single-pot solid phase enhanced sample preparation protocol [1]. Hydrophilic and hydrophobic Sera-Mag carboxylate-modified magnetic particles (GE Healthcare) were combined 1:1, washed two times with 10 volumes of water and stored at 10 µg/µl. 2 µl of the bead mix were added to the samples and acetonitrile (ACN) was added to a final percentage of 50%. The mixture was incubated at 25°C for 8 min with shaking at 1400 rpm. The plates were placed on a magnetic rack for 2 min, supernatants were removed and the beads were washed with 180 µl prewarmed isopropanol at 55°C and 1400 rpm for 10 min. Supernatants were removed on a magnetic rack and the isopropanol washes were repeated four more times. Finally, the trapped beads were washed with 200 µl acetonitrile on the magnetic rack, air dried and reconstituted in 50 µl 50mM Triethylammonium bicarbonate (TEAB) containing 1 µg Trypsin. Proteins were digested for 16 h at 37°C in a Thermocycler. After digestion samples were acidified with 50 µl 5 % formic acid. Beads were trapped on a magnetic rack for 3 min and 90 µl supernatant containing the digested proteins were transferred to fresh plates. 5 µl of each sample were diluted with 15 ul 0.1 % formic acid and loaded on Evotips (Evosep) according to the manufacturer instructions.

2. **Mass spectrometry**

   Samples were analyzed on a timsTOF Pro2 mass spectrometer (Bruker) that was equipped with a captive spray ion source and a fused silica emitter (20 µm inner diameter, Bruker) coupled with an Evosep One LC system. All samples and high pH fractions were analyzed using the predefined 30SPD method with a Performance 15 cm × 150 µm C-18 column (Evosep) that was maintained at 40°C in a column oven (Bruker). The buffer system consisted of 0.1 % formic acid in water (buffer A) and B 0.1 % formic acid in acetonitrile (buffer B). MS data were acquired in dia-PASEF mode. The method uses 16 scans with variable m/z windows and 2 ion mobility windows per scan to cover a mass range from 350 m/z to 1250 m/z and an ion mobility range from 1/K0 = 0.75 $V/cm^2$to 1/KO = 1.4 V/cm^2. Scan times were 100 ms, the resulting cycle time was 1.6 sec. The ion mobility was calibrated at the start of each run using the in-batch calibration option.

3. **Data processing**

   A predicted spectral library was generated using a human canonical Swissprot fasta file and DIA-NN 1.8.1 [2]. Settings for library generation were: Trypsin, up to 1 missed cleavage, precursor ion range 350 m/z – 1250 m/z, fragment ion range 200 m/z – 1700 m/z, charge states 2+, 3+ and 4+, peptide length 7, max peptide length 30 and cysteine carbamidomethylation enabled as a fixed modification. The predicted library was searched with the sample runs in match between run (MBR) mode and settings matching the library generation. Additional command line prompts were --report-lib-info and --relaxed-prot-inf. The DIA-NN output was further filtered on >= 4 fragment ions per precursor, library q-value <= 0.01, global q-value <= 0.01 and unique peptides using the dplyr package in R. Finally, LFQ values were calculated using the DIA-NN R-package.

4. **Data Analysis**

   The protein LFQ intensities were imported into Perseus 1.6.15.0 and filtered for at least 70% data completeness [3]. Missing values were imputed with random values from the lower end of the intensity distribution using Perseus default parameters.

Supplementary References

1. Hughes, C.S., Moggridge, S., Müller, T. *et al.* Single-pot, solid-phase-enhanced sample preparation for proteomics experiments. *Nat Protoc* **14,** 68–85 (2019). https://doi.org/10.1038/s41596-018-0082-x

2. Demichev, V., Messner, C.B., Vernardis, S.I. *et al.* DIA-NN: neural networks and interference correction enable deep proteome coverage in high throughput. *Nat Methods* **17,** 41–44 (2020). https://doi.org/10.1038/s41592-019-0638-x

3. Tyanova, S., Temu, T., Sinitcyn, P. *et al.* The Perseus computational platform for comprehensive analysis of (prote)omics data. *Nat Methods* **13**, 731–740 (2016). https://doi.org/10.1038/nmeth.3901
